# A Secure-Enhanced Data Aggregation Based on ECC in Wireless Sensor Networks

**DOI:** 10.3390/s140406701

**Published:** 2014-04-11

**Authors:** Qiang Zhou, Geng Yang, Liwen He

**Affiliations:** 1. Institute of Advanced Technology, Nanjing University of Posts & Telecommunications, Nanjing 210046, China; E-Mail: helw@njupt.edu.cn; 2. School of Computer Science & Technology, Nanjing University of Posts & Telecommunications, Nanjing 210046, China; 3. School of Computer & Information Engineering, Chuzhou University, Chuzhou 239000, China

**Keywords:** wireless sensor networks, data aggregation, Elliptic Curve Cryptography (ECC), data integrity, data privacy

## Abstract

Data aggregation is an important technique for reducing the energy consumption of sensor nodes in wireless sensor networks (WSNs). However, compromised aggregators may forge false values as the aggregated results of their child nodes in order to conduct stealthy attacks or steal other nodes' privacy. This paper proposes a Secure-Enhanced Data Aggregation based on Elliptic Curve Cryptography (SEDA-ECC). The design of SEDA-ECC is based on the principles of privacy homomorphic encryption (PH) and divide-and-conquer. An aggregation tree disjoint method is first adopted to divide the tree into three subtrees of similar sizes, and a PH-based aggregation is performed in each subtree to generate an aggregated subtree result. Then the forged result can be identified by the base station (BS) by comparing the aggregated count value. Finally, the aggregated result can be calculated by the BS according to the remaining results that have not been forged. Extensive analysis and simulations show that SEDA-ECC can achieve the highest security level on the aggregated result with appropriate energy consumption compared with other asymmetric schemes.

## 1. Introduction

Wireless sensor networks (WSNs) consist of thousands of sensors that collect data from a certain deployed range. Currently, WSNs have plenty of applications, such as military investigation, environment monitoring and accident reporting, *etc.* Typically, sensors have strictly limited computation and communication abilities and power resources; therefore, reducing the power consumption is a critical concern for WSNs. For better energy utilization, data aggregation [1,2] has been proposed recently. The original concept is to aggregate multiple sensing messages by performing statistical or algebraic operations, such as addition, minimum, maximum, median, *etc.* Since only the aggregated results need to reach the base station (BS) instead of sensing data, communication costs can be significantly reduced. Unfortunately, data aggregation is vulnerable to some attacks. For example, an adversary could compromise cluster heads (aggregators) similar to compromising all its cluster members. To solve this problem, several schemes, such as SDAP [3], PEPDA [4], Jung *et al.*'s scheme [5] have been proposed. However, these schemes can only guarantee the data privacy during the process of data aggregation and have a long aggregation delay.

An alternative method for secure data aggregation is to use privacy homomorphic encryption (PH), which can aggregate encrypted messages directly from sensors without decrypting so that it has a short aggregation delay. An adversary knows nothing from forging aggregated results even if the aggregators are compromised, because aggregators are unable to encrypt messages. PH is allowed to carry out specific types of computations on ciphertext, and the decrypted aggregation result matches the result of operations performed on the plaintext. PH has been used for data aggregation in WSNs, such as in Wang *et al.*'s scheme [6], CDAMA [7], Tiny PEDS [8], *etc.* However, the existing PH schemes suffer from the data integrity issue.

In this paper, we focus on bridging the gap between data privacy and integrity in WSNs. Some symmetric secure aggregation schemes [9,10] have been proposed to achieve both data privacy and integrity, but they cannot defend against node compromise attacks due to its inherent drawback that the encryption key is same as the decryption key. In general, symmetric schemes are less secure than asymmetric ones, although they are more efficient in terms of computational cost. Therefore, we originally propose a secure-enhanced data aggregation scheme based on Elliptic Curve Cryptography (ECC), called SEDA-ECC, which is an improved version of Boneh *et al.*'s asymmetric scheme [11]. To the best of our knowledge, SEDA-ECC can defend against the most attacks with appropriate energy consumption compared with other asymmetric schemes.

The rest of the paper is organized as follows: in Section 2, the existing secure data aggregation schemes in WSNs are presented. The system model and preliminaries are discussed in Section 3. In Section 4, a secure-enhanced data aggregation scheme based on ECC is proposed. Section 5 describes the security analysis of SEDA-ECC, and Section 6 presents performance evaluation and comparison to prove the effectiveness and efficiency of our scheme. Finally, we conclude SEDA-ECC in Section 7.

## 2. Related Works

Currently, many secure data aggregation schemes have been proposed. For symmetric schemes, Ozdemir *et al.* [9] integrated false data detection with data aggregation and confidentiality, and proposed an authentication protocol. In the scheme, every aggregator has some monitoring nodes which also perform data aggregation for data verification, and the integrity of the encrypted data is verified by the sensors between two consecutive aggregators. Its limitation is the rigorous topological constraints. Papadopoulos *et al.* [10] presented an exact aggregation scheme with integrity and confidentiality, named SIES. SIES combines the symmetric homomorphic encryption with secret sharing. A wide range of aggregates can be covered, and a small amount of bandwidth consumption is introduced in SIES. However, the data transmission efficiency is low due to the oversize space of secret keys. Based on Aggregation-Commit-Verify approach, Chan *et al.* [12] first proposed a provably secure hierarchical data aggregation scheme, where the adversary is forced to commit to its choice of aggregation results, then the sensors are allowed to verify whether their aggregation contributions are correct or not. The scheme can be used for multiple malicious nodes and arbitrary topologies, but it inherits the weakness of large amount of communication and computation overheads. To address this issue, Frikken *et al.* [13] improve Chan's scheme by reducing the maximum communication per node from *O*(Δlog^2^*n*) to *O*(Δlog*n*), where *n* is the number of nodes in WSNs, and Δ is the maximum degree of the aggregation tree.

For asymmetric schemes, Zhu *et al.* [14] focused on preserving data integrity and proposed an efficient integrity-preserving data aggregation protocol named EIPDAP. The scheme is based on the modulo addition operation using ECC, and has the most optimal upper bound on solving the integrity-preserving problem for data aggregation. Niu *et al.* [15] proposed a secure identity-based lossy data aggregation scheme using homomorphic hashing and identity-based aggregate signature. In the scheme, the authenticity of aggregated data can be verified by both aggregators and BS. The computation and communication overheads could be significantly reduced because the BS can perform batch verification. However, the above two schemes may lead to the leakage of data privacy due to decryption at the aggregator. Based on PH, Westhoff *et al.* [16] and Girao *et al.* [17] proposed CDA methods to facilitate aggregation in encrypted data, where richer algebraic operations can be directly executed on encrypted data by aggregators. Mykletun *et al.* [18] adopted several public-key-based PH encryptions to achieve data concealment in WSNs. Furthermore, Girao *et al.* [8] proposed a novel scheme by extending the ELGamal PH encryption. However, the above schemes cannot resist node compromise attacks. Specific security analysis is presented in Section 5.

## 3. System Model and Preliminaries

In this section, we describe the aggregation model and the attack model. The aggregation model defines how aggregation works, and the attack model defines what kinds of attacks our secure data aggregation scheme should protect against.

### 3.1. Aggregation Model

We consider large scale WSNs with densely deployed sensors. In WSNs, there are three types of nodes: base station (BS), aggregator, and leaf node. In this paper, we consider the aggregation tree roots at the BS like general data aggregation protocol [1,3]. Sensor nodes have overlapping sensing regions due to the dense deployment, and the same event is often detected by multiple sensors. Hence, data aggregation is proposed to reduce data transmission. The non-leaf nodes, except the BS, may also serve as aggregators. They are responsible for combining answers from their child nodes and forwarding intermediate aggregation results to their parents. Without loss of generality, we focus on additive aggregation, which can serve as the base of other statistical operations (e.g., count, mean, or variance).

### 3.2. Attack Model

First, we categorize the abilities of the adversary as follows:

(1) An adversary can eavesdrop on transmission data in a WSN.
(2) An adversary can send the forged data to leaf nodes, aggregators, or BS.
(3) An adversary can compromise secrets in sensors or aggregators.

Then, we define five attacks to qualify the security strength of the secure data aggregation schemes, based on adversary's abilities and purposes.

(1) Ciphertext analysis
  Ciphertext analysis is a very common and basic attack. In such an attack, an adversary wants to deduce the secret key or obtain information only by interpreting ciphertext. A secure scheme must ensure that it is not possible to gain any information or key, and an adversary cannot decide whether an encrypted ciphertext corresponds to a specific plaintext or not.
(2) Chosen plaintext attacks
  Given some chosen samples of plaintexts and corresponding ciphertexts, the adversary can determine secret information or deduce the key. A secure scheme must ensure that an adversary cannot deduce secret keys or additional information out of the known set, even with a large set of plaintexts and their ciphertexts.
(3) Malleability
  The aim of the adversary is to alter the valid ciphertexts without leaving marks. In this kind of attack, an attack can randomly generate meaningless ciphertexts that are syntactically correct to harm the system. For many PH schemes, it is possible to alter the ciphertexts without knowing the concrete content. Hence, a secure scheme should not let the adversary be able to successfully change the contents of encrypted packet.
(4) Unauthorized aggregation
  In this kind of attack, an adversary is to aggregate two or more ciphertexts into forged but format-valid ciphertexts, then to inject them into the network for vandalizing the system.
(5) Node compromise attacks

An adversary can compromise sensors or aggregators. When an adversary compromises an aggregator and gets its secret, it can easily launch unauthorized aggregation and malleability attacks. When an adversary compromises a sensor and gets its secret, it can decrypt the ciphertexts of all sensors in the symmetric schemes; besides, it also can impersonate the sensor or the other sensors to generate legal ciphertexts in both symmetric and asymmetric schemes.

### 3.3. Privacy Homomorphism

A privacy homomorphism is an encryption transformation which allows direct computation on the encrypted data. Let *m*_1_ and *m*_2_ be two plaintexts, and ⊗, × be the homomorphic operations on the ciphertexts and plaintexts respectively, we have Enc(*m*_1_) ⊗ Enc(*m*_2_) = Enc(*m*_1_ × *m*_2_), where Enc(*m*) represents the ciphertext of *m*. Component-wise multiplications and additions of ciphertexts result in the corresponding multiplications and additions of plaintexts. If *E*_(_*_p_*_,_*_q_*_)_(*m*_1_) = (*x*_1_,*y*_1_) and *E*_(_*_p_*_,_*_q_*_)_(*m*_2_) = (*x*_2_,*y*_2_), then:

(1) $$\begin{array}{ll} E_{\left(p,q\right)}(m_{1}+m_{2}) & =\text{Add}(E_{\left(p,q\right)}(m_{1}),E_{\left(p,q\right)}(m_{2}))\\ & =(x_{1}+x_{2}\mathrm{mod}n,y_{1}+y_{2}\mathrm{mod}n) \end{array}$$

(2) $$\begin{array}{ll} E_{\left(p,q\right)}(m_{1}m_{2}) & =\text{Multi}(E_{\left(p,q\right)}(m_{1}),E_{\left(p,q\right)}(m_{2}))\\ & =(x_{1}x_{2}\mathrm{mod}n,y_{1}y_{2}\mathrm{mod}n) \end{array}$$

However, symmetric cryptography-based privacy homomorphism has been proved to be insecure in chosen plaintext attacks for some specific parameters [19]. Therefore, privacy homomorphism based on asymmetric cryptography should be used instead of privacy homomorphism based on symmetric cryptography for some mission critical networks.

### 3.4. BGN Scheme

Boneh *et al.* [11] propose a PH scheme (abbreviated as BGN) based on the encryption schemes proposed by Paillier [20] and Okamoto-Uchiyama [21]. Both additive and multiplicative homomorphisms are provided in BGN, however, multiplicative homomorphism is inefficient and very expensive for WSNs because it is based on the bilinear pairing. Hence, we only adapt additive homomorphism of BGN to our scheme. The additive homomorphic encryption of BGN can be applied to private data aggregation, which is described in Algorithm 1.

Due to large computational overhead of the asymmetric cryptography, Boneh *et al.* construct BGN on a cyclic group of elliptic curve point. In phase 1 of BGN scheme, supposing *E* is the set of elliptic curve points that form a cyclic group, ord(*E*) denotes the number of points in *E*. Supposing 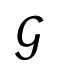 is a point in *E*, ord( 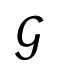) denotes the order of a point 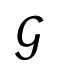. If ord( 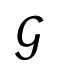) = *q*, there is q* 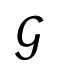 = ∞, where ∞ is the identity element of the group. In phase 2, point addition and scalar multiplication over points 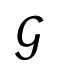 and 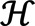 are used to encrypt the message *M*. Ciphertext *C* is composed of the message part and the secure randomness. In phase 3, BGN can aggregate the ciphertext due to homomorphic property. As we can see, the aggregated result will be the form of ΣM* 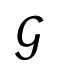 + ΣR* 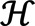, where Σ*M* is the sum of the messages, and Σ*R* is the sum of the randomness. In phase 4, BGN can decrypt the aggregated result to get the plaintext by multiplying the result with private key. When randomness of point 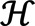 is removed by multiplying the order of 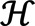, we can obtain ord( 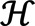;*ΣM* 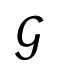. Finally, the plaintext Σ*M* can be retrieved by applying the discrete logarithm.

**Algorithm 1.** BGN scheme.Phase 1. Key-Gen(*λ*): generate a public-private key pair.01:Compute (*q_1_*, *q_2_*, *E*) using security parameter *λ*, where *E* is the set of elliptic curve points that form a cyclic group. ord(*E*)= *n*= *q_1_q_2_*, where *q_1_*, *q_2_* are large primes, and the bit lengths of them are the same, *i.e.*, |*q_1_*| = |*q_2_*|.02:Randomly select two generators, 

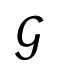
 and 

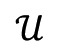
 such that ord(

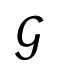
) = ord(

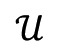
) = *n*.03:Compute point 

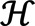
 = q_2_*

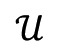
 such that ord(

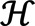
) = *q_1_*.04:Select parameter *T* < *q_1_* as the maximum plaintext boundary.05:Generate Public key *PK* = (*n*, *E*, 

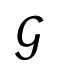
, 

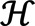
, *T*) and Private key *SK* = *q_1_*.Phase 2. Enc(*PK*, *M*): message encryption on *M* by public key *PK*.01:Check if the message space of a sensor node *M* ∈ {0, 1, …, *T*}.02:Random pick up *R* ∈ {0, 1, …, *n*−*1*}.03:Generate the ciphertext *C* =M*

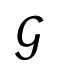
 + R*

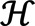
.04:Return *C*.Phase 3. Agg(*C_1_*, *C_2_*): message aggregation on two ciphertexts *C_1_* and *C_2_*, where *C_i_* = M_i_*

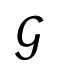
 + R_i_*

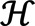
, *i* = 1,2.01:Randomly select *R*'∈{0, 1, …, *n* − *1*}.02:Compute the aggregated ciphertext *C′* = *C_1_* + *C_2_* + R′*

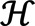
 = (*M_1_* + *M_2_*)*

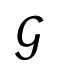
 + (*R_1_* + *R_2_* + *R′*)*

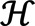
03:Return *C′*.Phase 4. Dec(*SK*, *C*): message decryption on *C* using private key *SK*.01:Compute *M* = log_
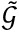
_ (*q*_1_ * *C*) log_
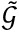
_ (*q*_1_ * (*M* *

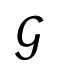
 + *R* * 

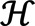
); = log_
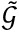
_ (*q*_1_ * *M* * 

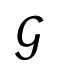
), where 

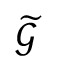
 = q_1_*

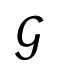
.02:Return *M*.

## 4. SEDA-ECC: A Secure-Enhanced Data Aggregation Based on ECC

In this section, we modify BGN to fit the SEDA-ECC scheme, so the security of BGN and SEDA-ECC are all based on the hardness assumption of subgroup decision problem. If we only provide the privacy protection of data aggregation, BGN can be used in SEDA-ECC directly, however, we also aim to ensure the data integrity, hence, different public-private key pairs and disjoint aggregation tree will be adopted. We first describe the details of SEDA-ECC scheme, which consists of six phases listed in Algorithm 2, then we present a case study of SEDA-ECC.

### 4.1. Key Generation Phase

Given a security parameter *λ*∈ℤ, the tuple (*q_1_*, *q_2_*, *q_3_*, *E*) is generated. *E* is the set of elliptic curve points that form a cyclic group, and ord(*E*) = *n* = *q_1_q_2_q_3_*, where *q_1_*, *q_2_*, *q_3_* are large primes, and the bit lengths of them are the same. Then, randomly select three points ( 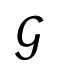
_1_, 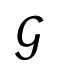
_2_, 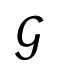
_3_) from *E*, where the order of 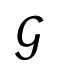
*_i_* is *n*, *i* = 1, 2, 3. Compute point 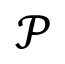 = q_2_q_3_* 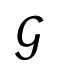
_1_, 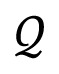 = q_1_q_3_* 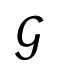
_2_, and point 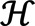 = q_1_q_2_* 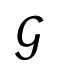
_3_, such that the order of 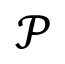, 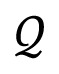 and 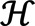 is *q_1_*, *q_2_*, and *q_3_* respectively.

**Algorithm 2.** SEDA-ECC scheme.Input:An aggregated WSN and SQL type SUM aggregation queryOutput:SUM aggregation result after integrity checked.Phase 1. Key-Gen(*λ*): generate public-private key pairs for tree *T_i_*, where *i* = *r*, *g* and *b*.01:Compute (*q_i_*_1_, *q_i_*_2_, *q_i_*_3_, *E*) based on security parameter *λ*, where *E* is the set of elliptic curve points that form a cyclic group. ord(*E*) = *n* = *q_i1_q_i2_q_i3_*, where *q_i1_*, *q_i2_*, *q_i3_* are large primes, and the bit lengths of them are the same, *i.e.*, |*q_i_*_1_| = |*q_i_*_2_| = |*q_i_*_3_|.02:Randomly select generators, 

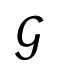
*_i_*_1_, 

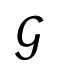
*_i_*_2_ and 

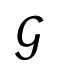
*_i_*_3_ such that ord(

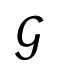
*_i_*_1_) = ord(

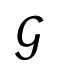
*_i_*_2_) = ord(

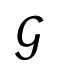
*_i_*_3_) = *n*.03:Compute point 

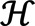
*_i_* = *q_i_*_1_*q_i_*_2_* 

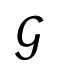
*_i_*_3_ such that ord(

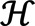
*_i_*) = *q_i_*_3_, 

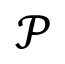
*_i_* = *q_i_*_2_*q_i_*_3_*

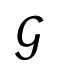
*_i_*_1_ such that ord(

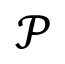
*_i_*) = *q_i_*_1_, and 

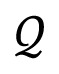
*_i_* = *q_i_*_1_*q_i_*_3_* 

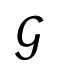
*_i_*_2_ such that ord(

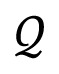
*_i_*) = *q_i_*_2_. Then output Public key *PK* = (*n*, *E*, 

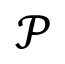
*_i_*, 

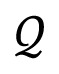
*_i_*, 

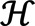
*_i_*) and Private key *SK* = {(*q_i_*_1_*q_i_*_3_), (*q_i_*_2_*q_i_*_3_)}.Phase 2. Dis-Tree(*p_r_*, *p_g_*, p*_b_*): disjoint aggregation tree construction with probability *p_r_*, *p_g_* and p*_b_*.01:BS triggers the aggregation by a *HELLO* message, when receiving such a message, nodes select their roles: red aggregator, green aggregator and blue aggregator. Aggregators then also forward the *HELLO* messages.02:If a node receives *HELLO* messages from red, green and blue aggregators, it randomly selects its role according to *p_i_*; otherwise it waits until the *HELLO* messages from all kinds of aggregators are received.03:Three disjoint aggregation trees rooted at the BS can be formed as the disjoint tree construction procedure continues. Red aggregators, green aggregators and blue aggregators interleave with each other.Phase 3. Enc(*PK*, *M_i_*): message encryption in three trees respectively, where *i* = *r*, *g* and *b*.01:Set *T_M_* < *q_i_*_1_. Check if the message space of a sensor node *M_i_* ∈ {0, 1, …, *T_M_*}.02:Randomly pick up *R_i_*∈{0, 1, …, *n* − *1*}.03:Generate the ciphertext *C_i_* = M_i_*

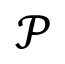

*_i_* + 

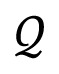
*_i_* + R_i_*

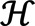
*_i_*.04:Return *C_i_*.Phase 4. Agg(*C_i_*_1_, *C_i_*_2_): message aggregation on two ciphertexts *C_i_*_1_ and *C_i_*_2_, where *i* = *r*, *g* and *b*.01:Compute the aggregated ciphertext *C_i_*_a_ = *C_i_*_1_ + *C_i_*_2_ = (Σ*M_ij_*)*

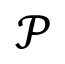
*_i_* + ζ_i_*

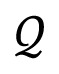
*_i_* + (Σ*R_ij_*)*

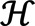
*_i_*, where Σ*M_ij_* represents the aggregated result of tree *T_i_*, ζ*_i_* represents the number of aggregated ciphertexts in tree *T_i_*, and Σ*R_ij_* represents the aggregated randomness in tree *T_i_*.02:Return *C_i_*_a_.Phase 5. Dec(*SK*, *C_i_*_a_): message decryption on *C_i_*_a_ in tree *T_i_*.01:Compute *M_i_* = Σ*M_ij_* = log**_
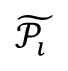
_***_l_* (*q_i_*_2_*q_i_*_3_), where 

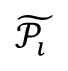
 = *q_i_*_2_*q_i_*_3_*

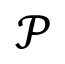
*_i_*.02:Compute ζ*_i_* = log**_
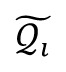
_***_l_*(*q_i_*_1_*q_i_*_3 *_
*C_i_*), where 

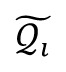
 = *q_i_*_1_*q_i_*_3_*

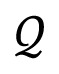
*_i_*.03:Return *M_i_*, ζ*_i_*.Phase 6. Chec(*M_i_*): check message *M_i_* integrity at the BS, where *i* = *r*, *g* and *b*.01:Set *i*, *j*,*k* ∈ {*r*, *g*, *b*}, and *i* ≠ *j* ≠ *k*.If |ζ*_i_* − ζ*_j_*| ≤ *Th*, and |ζ*_j_* − ζ*_k_*| ≤ *Th*,     BS accepts the three aggregated results and computes the final result *M* = *M_i_* + *M_j_* + *M_k_*;else if |ζ*_i_* − ζ*_j_*| ≤ *Th*, and |ζ*_j_* − ζ*_k_*| > *Th*,     BS rejects *M_k_*, and computes the final aggregated result *M* = 3/2(*M_i_* + *M_j_*);else if |ζ*_i_* − ζ*_j_*| > *Th*, and |ζ*_j_* − ζ*_k_*| > *Th*,     BS either decides which aggregated result is real through gathering topology information, or rejects all the aggregated result *M_i_*, *M_j_* and *M_k_*, and return NULL.

The scalar of 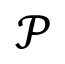 is the aggregated messages, the scalar of 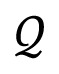 is the count of ciphertexts, and the scale of 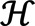 is randomness for security. We can check the integrity of the aggregated results by its count, the detail of check method is described in phase 6. For each subtree, the Public key is *PK* = (*n*, *E*, 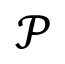, 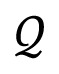, 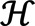 and the Private key is *SK* = {(*q_1_q_3_*), (*q_2_q_3_*)}.

### 4.2. Aggregation Tree Disjoint Phase

**Figure 1.**
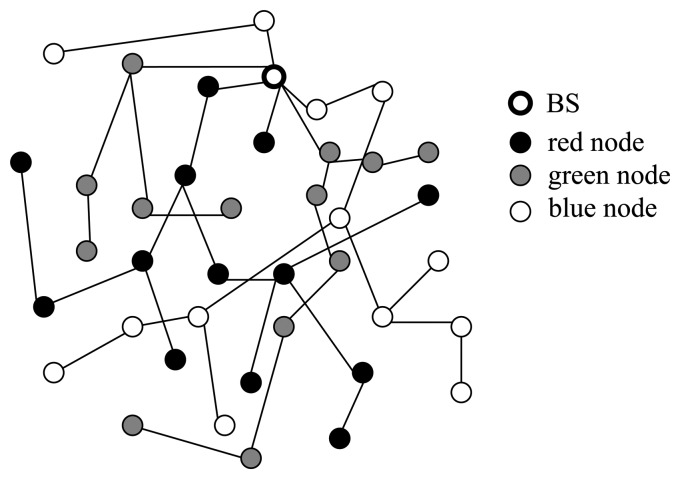
Disjoint tree construction.

Three subtrees are built in this scheme, which are called red aggregation tree, green aggregation tree, and blue aggregation tree, respectively, and the BS is the root of the above three subtrees. Assuming the network is dense enough, each node, except the BS, takes one of the four roles: red aggregator, green aggregator, blue aggregator, or leaf node. We partition the tree into three subtrees, the disjoint tree is as shown in Figure 1, where the black colored nodes represent red aggregators, grey colored nodes represent green aggregators, and white colored nodes represent blue aggregators.

#### Step 1

BS is appointed to be the root of the above three subtrees, which initiates a *HELLO* message requesting sensors to organize into one of the three aggregation trees. In that message, it contains its own ID and its level information *L_r_* = *L_g_* = *L_b_* = 0.

#### Step 2

Each sensor receiving the message should make the decision on its role, assign its own level to be *L_i_* + 1(*i* = *r*, *g*, *b*), and select the sender node as its parent. A node becomes a red aggregator with probability *p_r_*, a green aggregator with probability *p_g_*, and a blue aggregator with probability *p_b_*, respectively. The probability will be subject to the conditions: 0 < *p_r_* = *p_g_* = *p_b_* < 1, and *p_r_* + *p_g_* + *p_b_* = 1.

#### Step 3

Each node in one aggregation tree rebroadcasts the colored message corresponding tree, which contains its own ID and level. If any node has already been in the tree when receives the message, it will reject the message; otherwise, the node also assigns its level *L_i_* to be *L_i_* + 1. Three aggregation trees are constructed till all nodes have a level and a parent. To balance the red, green and blue aggregators in a given neighborhood, a node should wait enough time to receive *HELLO* messages from red, green and blue aggregators as much as possible before the decision on its color is made. Then, *p_r_*, *p_g_* and *p_b_* can be computed by each node as follows:

(3) $$p_{r}=\frac{1}{2}\cdot \frac{N_{g}+N_{b}}{N_{r}+N_{g}+N_{b}},p_{g}=\frac{1}{2}\cdot \frac{N_{r}+N_{b}}{N_{r}+N_{g}+N_{b}},p_{b}=\frac{1}{2}\cdot \frac{N_{r}+N_{g}}{N_{r}+N_{g}+N_{b}}$$

where *N_i_* is the number of *HELLO* messages that one sensor receives from the *i* aggregators (*i* = *r*, *g*, *b*). It should be noted that only a very few nodes do not participate in data aggregation when the network is dense enough.

#### Step 4

During the process of aggregation, red aggregators are not allowed to forward the data for green and blue aggregators, and *vice versa*. Then, the separation of data aggregation can be achieved along the disjoint trees. Finally, the BS will receive three aggregated results *M_r_*, *M_g_* and *M_b_* respectively.

Note that an adversary may compromise the data integrity during this phase by sending two *HELLO* messages with different colors. This can be prevented by guaranteeing that a node in one tree cannot be in another two trees. However, such attack can be detected easily by its neighbors because of the shared-medium nature of wireless links. Therefore, the adversary can be excluded from the three aggregation trees.

### 4.3. Encryption Phase

We set *T_M_* < *q_1_*. The message space of a sensor node *M* should subject to *M_i_* ∈ {0, 1, …, *T_M_*}, where *i* = *r*, *g* and *b*. Each sensor picks a random *R_i_* ∈ {0, 1, …, *n* − 1}, and encrypt the message *M_i_* using public key *PK*, then it generates the ciphertext *C_i_* = M_i_* 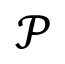 + 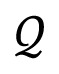 + R_i_* 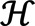, where + is the addition of elliptic curve points and * is the scalar multiplication of elliptic curve.

### 4.4. Aggregation Phase

Let Σ*M_ij_* denote the aggregated message of tree *T_i_*, ζ*_i_* denote the number of aggregated ciphertexts of tree *T_i_*, and Σ*R_ij_* denote the aggregated randomness in tree *T_i_*, consequently, *k* ciphertexts for *j* = 1 to *k* are aggregated into a ciphertext of *C_i_*_a_ as follows:

(4) $$C_{i\text{a}}=(\sum_{j=1}^{k}M_{ij})*P+\xi_{i}*Q(\sum_{j=1}^{k}R_{ij})*H$$

### 4.5. Decryption Phase

During the decryption phase, the BS can separately decrypt the aggregated result *M_i_* and its count ζ*_i_* from the aggregated ciphertext in tree *T_i_* respectively as follows:

(5) $$M_{i}=\sum M_{ij}=\log_{\tilde{P}}(q_{2}q_{3}*C_{i}),\;\text{where}\;\tilde{P}=q_{2}q_{3}*P$$

(6) $$\xi_{i}=\log_{\tilde{Q}}(q_{1}q_{3}*C_{i}),\;\text{where}\;\tilde{Q}=q_{1}q_{3}*Q$$

### 4.6. Data Integrity Check Phase

When the BS receives the three aggregated results from the red, green and blue subtrees, it should decrypt them and extract the count ζ*_i_*, respectively. If a compromised aggregator tampers with the aggregated result M_i_, the count value ζ*_i_* must be changed simultaneously because the aggregators do not know the base point 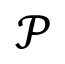 and 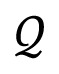. Therefore, the BS will compare ζ*_i_* with each other, and it indicates that the messages have not been tampered with en route only if they are almost the same. We set *Th* as difference threshold parameter, *i*, *j*, *k* ∈ {*r*, *g*, *b*}, and *i* ≠ *j* ≠ *k*.

If |ζ*_i_* − ζ*_j_*| ≤ *Th*, and |ζ*_j_* − ζ*_k_*| ≤ *Th*, it shows each result has not been tampered, then the BS accepts the three aggregated results and computes the final result *M* = *M_i_* +*M_j_* + *M_k_*; if |ζ*_i_* − ζ*_j_*| ≤ *Th*, and |ζ*_j_* − ζ*_k_*| > *Th*, it shows *M_k_* has been tampered, then the BS rejects *M_k_*, and computes the approximate aggregated result = 3/2(*M_i_* + *M_j_*); if |ζ*_i_* − ζ*_j_*| > *Th*, and |ζ*_j_* − ζ*_k_*| > *Th*, it shows the three aggregated results maybe have been tampered totally, then the BS either rejects all the aggregated results *M_i_*, *M_j_* and *M_k_*, or decides which aggregated result is real by gathering topology information.

### 4.7. A Case Study

**Figure 2.**
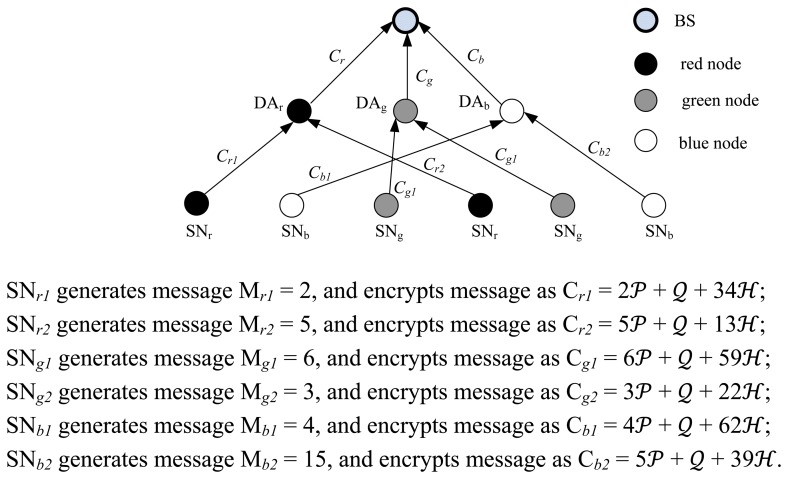
A case study of SEDA-ECC.

We present a case study to show how SEDA-ECC works. For simplicity, we assume that the network only consists of six leaf nodes and three aggregators besides BS, and the three subtrees have the same public key *PK* = (*n*, *E*, 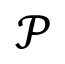, 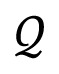, 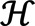);. As shown in Figure 2, each subtree has two sensor nodes and one aggregator. Three aggregators, DA*_r_*, DA*_g_* and DA*_b_* are deployed to gather messages from their child nodes respectively. For simplicity, the order of 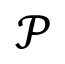, 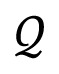 and 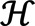 are set to small numbers. Supposing the order of 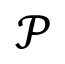 and value of *q_1_* is 13, the order of 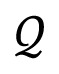 and value of *q_2_* is 17, and the order of 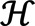 and value of *q_3_* is 19, then the order of *n* = *q_1_q_2_q_3_* is 4,199. Sensors in three subtrees encrypt and send their data as follows, where the scalars of 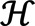 are randomly generated by sensors.

The encrypted messages are sent to data aggregators. Data aggregator DA*_r_* aggregates C*_r1_* and C*_r2_* as C*_r_* = 7 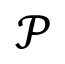 + 2 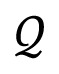 + 47 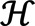. Similarly, data aggregator DA*_g_* aggregates C*_g1_* and C*_g2_* as C*_g_* = 9 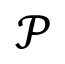 + 2 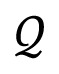 + 81 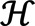, data aggregator DA*_b_* aggregates C*_b1_* and C*_b2_* as C*_b_* = 9 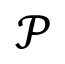 + 2 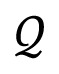 + 101 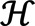. Because the order of 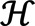 is 19, 19 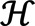 = ∞, where ∞ is the additive unit element in ECC. Therefore, we can get C*_r_* = 7 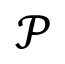 + 2 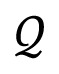 + 9 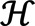, C*_g_* = 9 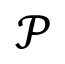 + 2 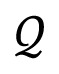 + 5 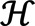, and C*_b_* = 9 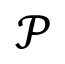 + 2 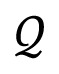 + 6 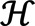.

The aggregated result of red subtree *M_r_* = *M_r1_* + *M_r2_* = 7 can be obtained by decrypting C*_r_* as follows:

(1) Compute q_2_q_3_* C*_r_* = 323* (7 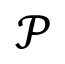 + 2 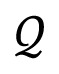 +9 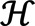) = 2,261 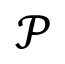 = 12 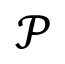, where 13 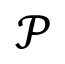 = 17 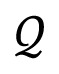 = 19 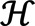 = ∞.
(2) *M_r_* = log**_
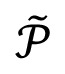
_**(*q*_2_*q*_3_ * *C_r_*)= log**_
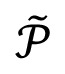
_** 12 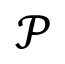, where 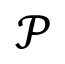
= q_2_q_3_* 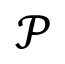 = 323 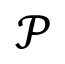 = 11 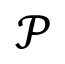. Then *M_r_* * 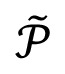 = M_r_*11 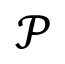 = 12 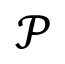 can be obtained because *M_r_* = log_
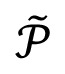
_ 12 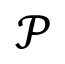.
(3) Finally, the aggregated result of red subtree *M_r_* = 7 can be obtained by the BS according to Pollard's *λ* method.

Similarly, the BS can also extract the aggregated count result ζ by computing the discrete logarithm of q_1_q_3_*C*_r_* to the base point 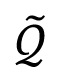 = q_1_q_3_* 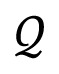. Therefore, BS can identify the forged result by comparing the aggregated count value. If the difference among three subtrees aggregated results is within the range of threshold *Th*, then BS validates the integrity of the aggregated result.

## 5. Theoretical Analysis

In this section, we analyze the coverage of aggregation trees first because it has great effect on our scheme's availability, then analyze the security of SEDA-ECC and compare it with five well-known secure data aggregation schemes: CDA [16,17], Castelluccia *et al.*'s scheme [22], BGN scheme [11], EC-OU scheme [18], and TinyPEDS scheme [8].

### 5.1. Coverage of Aggregation Trees

In SEDA-ECC scheme, a sensor reports its data to BS by aggregation only when it can reach red, green and blue aggregation trees within one hop. If a node cannot reach the three aggregation trees, it is disconnected from the BS for aggregation. We define Φ(*G*) as the probability that all the sensors are covered by all the three aggregation trees. It means that many sensors cannot contribute their data to the aggregation result if Φ(*G*) is small. Therefore, the coverage of aggregation trees impacts the accuracy of aggregation results. The aggregation accuracy is one of the most important performance metrics, because it can affect the decision of BS, so we should first analyze the coverage of aggregation trees to verify our scheme's availability.

Consider a random network *G* (*n*, *l*), where *n* is the number of sensors, and *l* is the transmission range of a sensor. We randomly assign red, green or blue to sensors in the networks, and let *S* denote the number of sensors which are isolated from red, green or blue sensors, then:

(7) Φ(G)=P(S=0)

We define *S_i_* as the variable of whether sensor *i* has red, green and blue neighbors within one hop distance, then

(8) $$S_{i}=\{\begin{array}{ll} 0, & i\;\text{has red},\text{green and blue neighbors}\\ 1, & \text{otherwise} \end{array}$$

{*S_i_*} can be approximated as identical independent distributions for a random network whose size is large enough, therefore, $S=\sum_{i=1}^{n}S_{i}$ can be denoted as the total number of sensors which are isolated by red, green or blue aggregation tree. Let *d_i_* denote the number of neighbors of sensor *i*, then the probability that *i* is isolated by the red aggregation tree is labeled as $p_{bg}^{d_{i}}$. Similarly, *i* is isolated by the green (blue) aggregation tree with the probability $p_{rb}^{d_{i}}(p_{rg}^{d_{i}})$. Let *p_i_* be the probability that note *i* is isolated by red sensors, green sensors or blue sensors, then:

(9) $$p_{i}=P(S_{i}=1)=1-(1-p_{bg}^{d_{i}})(1-p_{rb}^{d_{i}})(1-p_{rg}^{d_{i}})$$

Since $S=\sum_{i=1}^{n}S_{i}$, we can get a lower bound of Φ(*G*) by applying Markov Inequality $P(S\geq 1)\leq E[S]=\sum_{i=1}^{n}p_{i}$. That is:

(10) $$\Phi (G)\geq \frac{n-\sum_{i=1}^{n}p_{i}}{n}$$

**Figure 3.**
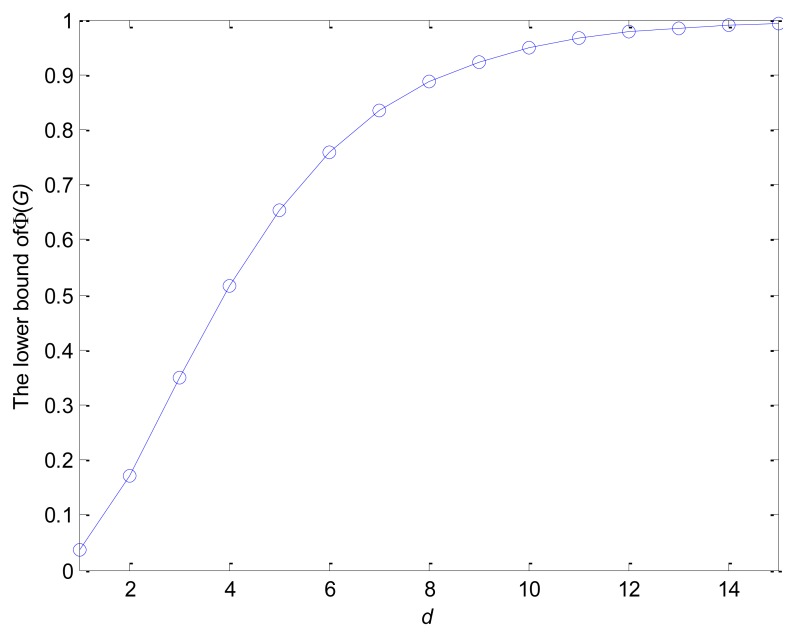
The lower bound of Φ(*G*) which varies with the variation of *d*.

When the network is dense enough, *i.e.*, *d_i_* is large, a small *p_i_* can be obtained. For example, assuming *p_r_* = *p_g_* = *p_b_* = 1/3, we can obtain the lower bound of Φ(*G*) which varies with the variation of *d* under the condition of the *d*-regular network according to Equation (9). It can be observed from Figure 3 that Φ(*G*) ≥ 0.95 for *d* = 10, therefore, the coverage of aggregation trees is perfect for dense networks from Equation (10).

### 5.2. Ciphertext Analysis

This is the most basic attack in WSNs. SEDA-ECC is robust to ciphertext analysis attack, because the elliptic curve cryptography-based encryption depends on the factorization of large integers. Other schemes are also robust to ciphertext analysis attacks.

### 5.3. Chosen Plaintext Attacks

SEDA-ECC is robust to chosen plaintext attacks, because its encryption relies on random numbers, and the ciphertext is probabilistic. Other schemes based on ECC can defend against this attack too. Wagner's cryptanalysis [23] has indicated that CDA might suffer from chosen plaintext attacks because of improper security parameters. However, the cost of proper parameter would render CDA infeasible to WSNs. Castelluccia *et al.*'s scheme is also robust to this attack, because its security is based on the indistinguishability property of a pseudorandom function, and the previous encryption keys cannot be used to deduce the present or subsequent encryption key.

### 5.4. Malleability

In the analysis of this attack, we give the example that the adversary wants to increase the measured data by 50. Since Castelluccia *et al.*'s scheme is based on modular addition, adversaries can add the value of plaintext trivially through adding a certain value to the corresponding ciphertext directly, so it suffers from this attack. For example, a ciphertext (*m* + *K_n_*) mod *M* can be easily altered by ((*m* + 50) + *K_n_*) mod *M* = (*m* + *K_n_*) + 50 mod *M*. Other schemes can defend against this attack because they are based on either modular multiplication or ECC.

### 5.5. Unauthorized Aggregation

For asymmetric scheme, SEDA-ECC, BGN, EC-OU and TinyPEDS are based on ECC. If an aggregator needs to perform aggregation, it has to know curve information. Since the public key is preinstalled in sensors generally, adversary cannot perform unauthorized aggregation and falsify the aggregated count value of subtrees without compromising the sensors or aggregators. CDA and Castelluccia *et al.*'s scheme might suffer from this attack, because they require only modular addition, and unauthorized aggregation can be performed without any additional information.

### 5.6. Node Compromise Attacks

For asymmetric schemes, SEDA-ECC, BGN, EC-OU and TinyPEDS do not suffer from unauthorized decryption under compromised sensor node conditions, because an adversary cannot obtain the private key through a compromised sensor. However, except for SEDA-ECC, they cannot defend against unauthorized aggregation in a compromised aggregator situation. The compromised aggregator might arbitrarily increase the aggregated result by aggregating the same ciphertext repeatedly or decrease it by selective aggregation. After the aggregation process, the forged value is difficult to detect or remove by the BS. SEDA-ECC can prevent this attack targeting data integrity by constructing disjoint aggregation subtrees. It is impossible for attackers to alter the aggregated result *M* without changing the count value ζ because the aggregators do not know the base points 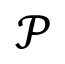 and 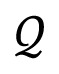. If the aggregated result of one tree is different from the others, the BS will reject it and compute the final result from the others. Therefore, an attacker can successfully forge the aggregated result if and only if the forged aggregated results of two trees are the same. The probability of success is extremely small, because the security depends on the factorization of large integers.

We use the case study of SEDA-ECC in Section 4.7 to validate its ability of defending against this attack. Supposing the aggregation ciphertexts excluding C*_r_*, C*_g_*, and C*_b_* are C'*_r_* = *M*'*_r_* 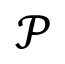 + 193 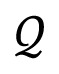 + *R_r_* 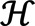, C'*_g_* = *M*'*_g_* 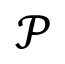 + 190 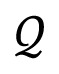 + *R_g_* 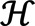, and C'*_b_* = *M*'*_b_* 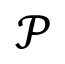 + 191 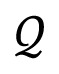 + *R_b_* 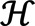. If the red aggregator DA*_r_* is compromised, it can arbitrarily increase the aggregated result by aggregating the same ciphertext repeatedly. Supposing the compromised aggregator DA*_r_* intend to increase C*_r_* by aggregating C*_r_*_1_ 20 times, then C*_r_* = 20C*_r_*_1_ + C*_r_*_2_ = 45 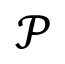 + 21 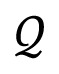 + 693 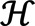. Therefore, we can get the aggregation ciphertext results ℂ*_r_* = C'*_r_* + C*_r_* = (*M*'*_r_* + 45) + 214 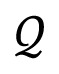 + (*R_r_* + 693) 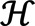, ℂ*_g_* = C'*_g_* + C*_g_* = (*M*'*_g_* + 9) 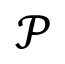 + 192 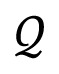 + (*R_g_* + 5) 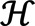), and ℂ*_b_* = C'*_b_* +C*_b_* = (*M*'*_b_* + 9) 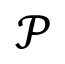 + 193 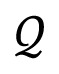 + (*R_b_* + 6) 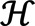, respectively. When the aggregated count results (r*_r_*, ζ*_g_*, ζ*_b_*) are extracted by computing the discrete logarithm of *q*_1_*q*_3_*(ℂ*_r_*,ℂ*_g_*,ℂ*_b_*) to the base point 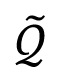
= *q*_1_*q*_3_* 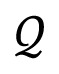, the forged result ℂ*_r_* can be easily identified and rejected by BS because the differences between ζ*_r_* and the other two are out of the threshold value *Th*, that is |ζ*_r_* − ζ*_g_*| = 22 > *Th*, and |ζ*_r_* – ζ*_b_*| = 21 > *Th*.

For symmetric schemes, the inherent drawback of CDA and Castelluccia *et al.*'s schemes is that the encryption key is identical with the decryption key. Therefore, an adversary can decrypt the ciphertext once the sensor is compromised. In addition, because the CDA's key is shared by all sensors and BS, if any sensor is compromised, the whole system security is broken. Castelluccia *et al.*'s scheme suffers from a minor impact due to the fact its distinct key is shared by BS.

Table 1 shows the security analysis comparisons for all schemes. It clearly shows that symmetric schemes are less secure than asymmetric ones, although they are more efficient in terms of communication and computation costs. Compared with other asymmetric schemes, SEDA-ECC is superior in defending against compromised node attacks because it can protect data integrity by constructing disjoint aggregation trees when the aggregators are compromised.

## 6. Performance Evaluation and Comparison

**Figure 4.**
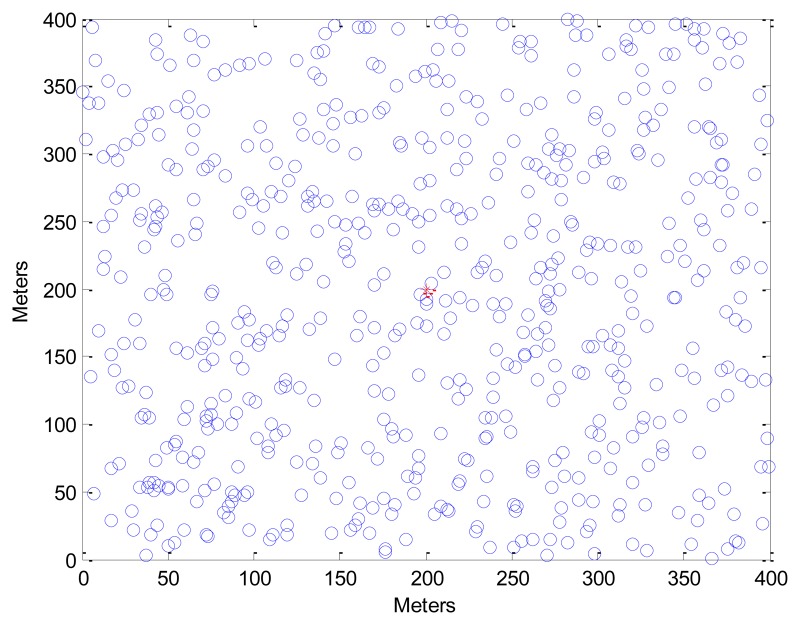
Nodes distribution.

Generally, symmetric key-based homomorphic schemes are more efficient than asymmetric ones, however, the security of symmetric schemes is weaker than that of asymmetric ones. For the sake of fairness, the performance of SEDA-ECC is only compared with other three asymmetric key-based homomorphic encryption schemes. In this section, we first discuss the threshold value *Th*, then evaluate the computation overhead, communication cost, and the accuracy of SEDA-ECC, BGN, EC-OU and TinyPEDS. We conduct simulations using TinyOS 2.0 simulator (TOSSIM). The parameters are shown in Table 2, and the topology of nodes is depicted in Figure 4, where the transmission range of a sensor is 50 m, and the BS coordinate is (200,200).

### 6.1. Th Parameter Setting

In general, the more sensors that participate in the data aggregation, the larger the probability of constructing disjoint aggregation trees which have the same number of sensors. In addition, the aggregated count results ζ from three aggregation trees may not agree with each other exactly due to collisions and congestions in wireless channels. Therefore, an adjustable threshold value *Th* and the lowest bound of network size are introduced to accomodate these factors. Since whether the BS accepts the result depends on the threshold value *Th*, hence *Th* is an important parameter. In order to get *Th*, we did extensive simulations, where the number of nodes (network size) was varied from 300 to 1,200 in a 400 m × 400 m area.

**Figure 5.**
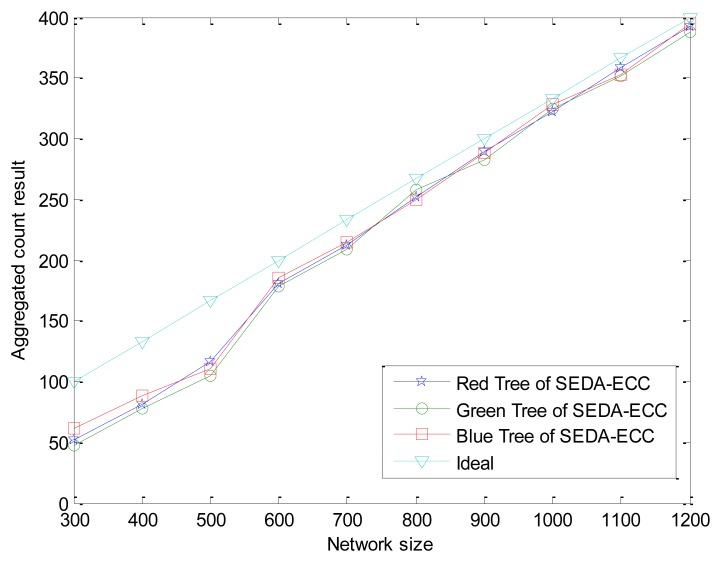
Difference value among aggregated count results from three subtrees without attack.

The difference value among aggregated count results from three aggregation subtrees is simulated 40 times, and the average value is depicted in Figure 5, where the “ideal” curve shows the aggregated result in an ideal situation. According to the simulation result, we notice that the differences, which are between 2 and 9, are very small. Hence, the threshold can be set as a small value, e.g., *Th* = 10. We can adjust *Th* if the network conditions are changed. Note that the average count result is only half of the ideal number and the difference extends to 9 when the network size is nearly 300. In addition, the smaller network size is, the larger differences became. As we analyzed in Section 5.1, it is because the coverage is bad enough in a sparse network to deteriorate the aggregation accuracy. Therefore, we set the lowest bound of network size as 300 in a 400 m × 400 m area to make our scheme available.

### 6.2. Communication Overhead

**Figure 6.**
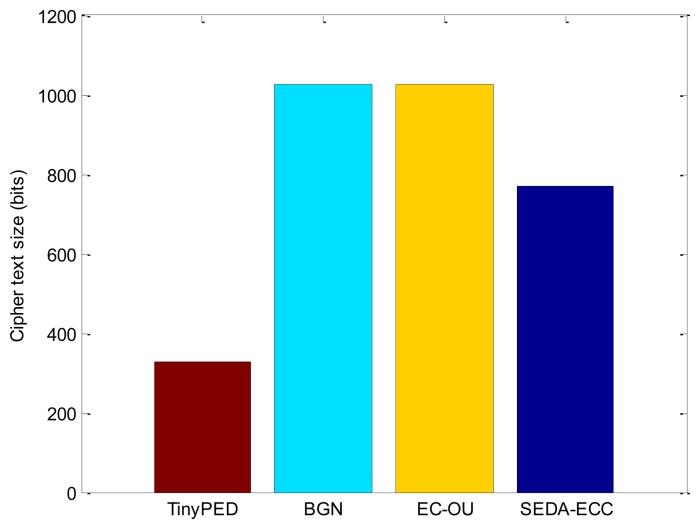
The comparison of ciphertext sizes.

The number of exchanged messages in each scheme is the same. Though there are three subtrees need to be built in SEDA-ECC, similar to the other schemes, each node needs to send two messages for data aggregation: one *HELLO* message to form the aggregation tree, and the other message for data aggregation. Therefore, the communication overhead mainly depends on the ciphertext size of each scheme on the condition that the number of message sending to the BS is the same. Supposing the order of elliptic curve is *N*, SEDA-ECC's security relies on the hardness of factoring the order *N. N* is a product of several different large prime numbers, e.g., *N* = *q*_1_*q*_2_⋯*q_k_*, where *k* is the number of prime numbers. If the length of prime number is 256-bit, there is no efficient approach to factor the product *N* [7]. Therefore, in SEDA-ECC, we generate *N* = *q*_1_*q*_2_*q*_3_, where the prime numbers *q_i_* are all 256-bit. Since the size of the ciphertext is almost the same as |*N*| + 1, the SEDA-ECC's ciphertext size is 3 × |*q*| + 1(|*q*| = 256-bit). EC-OU's ciphertext size is 3 × |*q*| + 2(|*q*| = 341-bit) according to [24]. BGN's ciphertext size is 1,025-bit, and TinyPEDS's ciphertext size is 328-bit according to [7]. Figure 6 shows the comparison of ciphertext sizes.

### 6.3. Computation Overhead

Since SEDA-ECC, BGN, EC-OU and TinyPEDS schemes are all built on elliptic curves, encryption and aggregation operation are based on point addition and point scalar multiplication. In elliptic curve arithmetic, point doubling and adding are two basic operations. Scalar multiplication can be accomplished by the half-and-add algorithm based on point doubling and adding [25]. It requires about |*r*| doubling and |*r*|/2 additions for computing r* 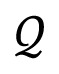, amounting to around 3|*r*|/2 point additions [18].

**Figure 7.**
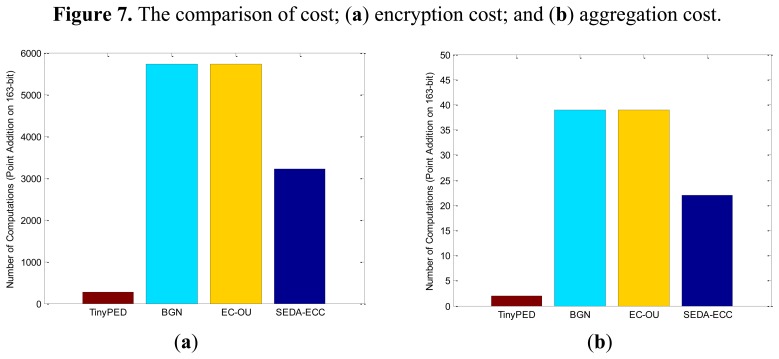
The comparison of cost; (**a**) encryption cost; and (**b**) aggregation cost.

It should be noted that SEDA-ECC, BGN, EC-OU and TinyPEDS schemes are built on different mathematical foundations. We assume the finite field of elliptic curve is 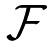
*_p_*, and the bit length of the finite field is |*p*|. BGN and EC-OU schemes are chosen over 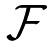
*_p_* (|*p*| = 1,024), TinyPEDS is chosen over 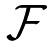
*_p_* (|*p*| = 163), SEDA-ECC is chosen over 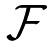
*_p_* (|*p*| = 768). To achieve a fair comparison, we choose the point addition on 163-bit field as the base unit. For an elliptic curve computation over a finite field 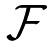
*_p_*, the cost of scalar multiplication can be converted to the number of computations (point addition on 163-bit) according to the scalar *r* and the size |*p*|. The comparison results are presented in Figure 7, where the length of messages is 16-bit, and the length of random nonces is 80-bit.

In summary, TinyPED is the most efficient one for both communication overhead and computation cost, because its curves are chosen from the smaller field 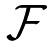
*_p_* (|*p*| = 163). TinyPED's security is based on the hardness of elliptic curve discrete logarithm problem, hence it can be built on a smaller field. However, BGN, EC-OU and SEDA-ECC are all based on the hardness of integer factorization problem, so their curves must be chosen from the larger field. It can also be observed from Figures 6 and 7 that SEDA-ECC outperforms BGN and EC-OU for both communication and computation performances. Furthermore, In terms of security, SEDA-ECC can defend against all attacks which are listed in Table 1, hence it is superior to the other schemes.

**Figure 8.**
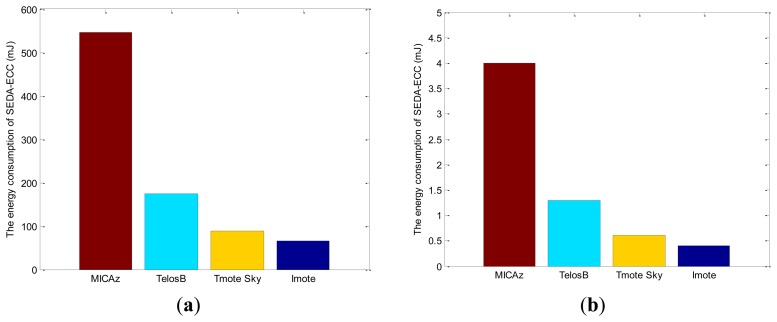
The energy consumption of SEDA-ECC in different sensor devices; (**a**) encryption consumption and (**b**) aggregation consumption.

Furthermore, the energy consumption of SEDA-ECC is evaluated in different sensor devices according to TinyECC [26], which is one well-known implementation of ECC for WSNs, as shown in Figure 8. The energy consumption can be significantly reduced with more advanced devices. Therefore, the secure data aggregation schemes based on asymmetric encryption, e.g., ECC, have extensive applications with the development of the advanced sensors.

### 6.4. Accuracy

**Figure 9.**
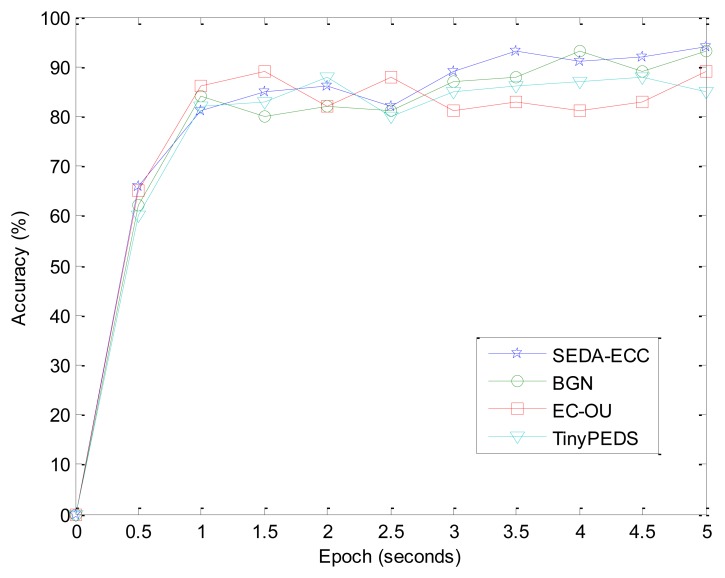
The comparison of accuracy with respect to different time intervals.

We define the accuracy as the ratio between the aggregated sum result by the data aggregation scheme in use and the real sum of all sensors participating in the data aggregation. It is an important issue because it could affect the decision of the BS. All the schemes should achieve 100% accurate aggregated results in an ideal situation. However, data packets may be lost or delayed due to data collisions, processing delays and noisy wireless channels. We evaluate the data accuracy of SEDA-ECC, BGN, EC-OU, and TinyPED with respect to different time intervals, as shown in Figure 9.

It shows that all these schemes almost perform equally in term of accuracy. We can observe that the accuracy increases as the time interval increases, because the data collisions and congestions between data aggregators are reduced, and the data packets should have enough time to be delivered.

## 7. Conclusions

Providing hierarchical data aggregation without losing data privacy and integrity guarantee is a challenging problem in WSNs. In this article, we propose a novel Secure-Enhanced Data Aggregation based on Elliptic Curve Cryptography (SEDA-ECC) for WSNs. SEDA-ECC divides the aggregation tree into three subtrees to reduce the importance of the high-level sensor nodes. It also generates three aggregated results by performing PH-based aggregations in the three subtrees, respectively, so that the BS could verify the subtree aggregated results by comparing the aggregated count value. Extensive analytical and simulation results indicate that SEDA-ECC can achieve the highest security level on the aggregated result comparing with other asymmetric schemes, and SEDA-ECC is efficient with respect to a reasonable energy cost.

## Figures and Tables

**Table 1.** Security comparisons.

**Requirement**	**Ciphertext Analysis**	**Chosen Plaintext Attacks**	**Malleability**	**Unauthorized Aggregation**	**Node Compromise Attacks**
CDA	√	×	√	×	×
Castelluccia *et al.*'s scheme	√	√	×	×	×
SEDA-ECC	√	√	√	√	√
BGN	√	√	√	√	×
EC-OU	√	√	√	√	×
TinyPEDS	√	√	√	√	×

**Table 2.** Simulation parameters.

**Radio Parameters**		**Topology Parameters**	

**Noise Floor**	**White Gaussian Noise**	**Terrain Dimensions**	**Number of Nodes**
			
−105 dB	4 dB	400 m × 400 m	600
